# Behavioral Differences between Male and Hermaphrodite *C. elegans*

**DOI:** 10.17912/micropub.biology.000431

**Published:** 2021-07-30

**Authors:** Elizabeth Loxterkamp, Jaaram Cha, Katharine Wu, Janessa Sullivan, Olivia Holbrook, Hazar Ghaith, Lena Srun, Deborah E. Bauer

**Affiliations:** 1 Department of Neuroscience, Wellesley College, Wellesley, Massachusetts, USA; 2 Department of Biological Sciences, University of Southern California, Los Angeles, California, USA

## Abstract

*C. elegans *are microscopic nematodes used extensively as a model organism due to their simplicity, allowing researchers to study basic molecular processes in biology. Most *C. elegans* are hermaphrodites, possessing two X chromosomes and the ability to reproduce asexually, but approximately 0.1% are males, arising due to a spontaneous loss of an X chromosome. In order to evaluate the behavioral sex differences in *C. elegans*, we expanded upon existing literature and compared spontaneous movement, sensitivity to mechanosensation, and sensitivity to chemosensation between males and hermaphrodites. In our paradigms, we found that males and hermaphrodites exhibit similar spontaneous movement as well as similar slow and sustained behaviors such as chemotaxis, but differ in quick-response to mechanical and chemosensory stimuli.

**Figure 1. Behavioral Test Results Between Male and Hermaphrodite C. elegans:**
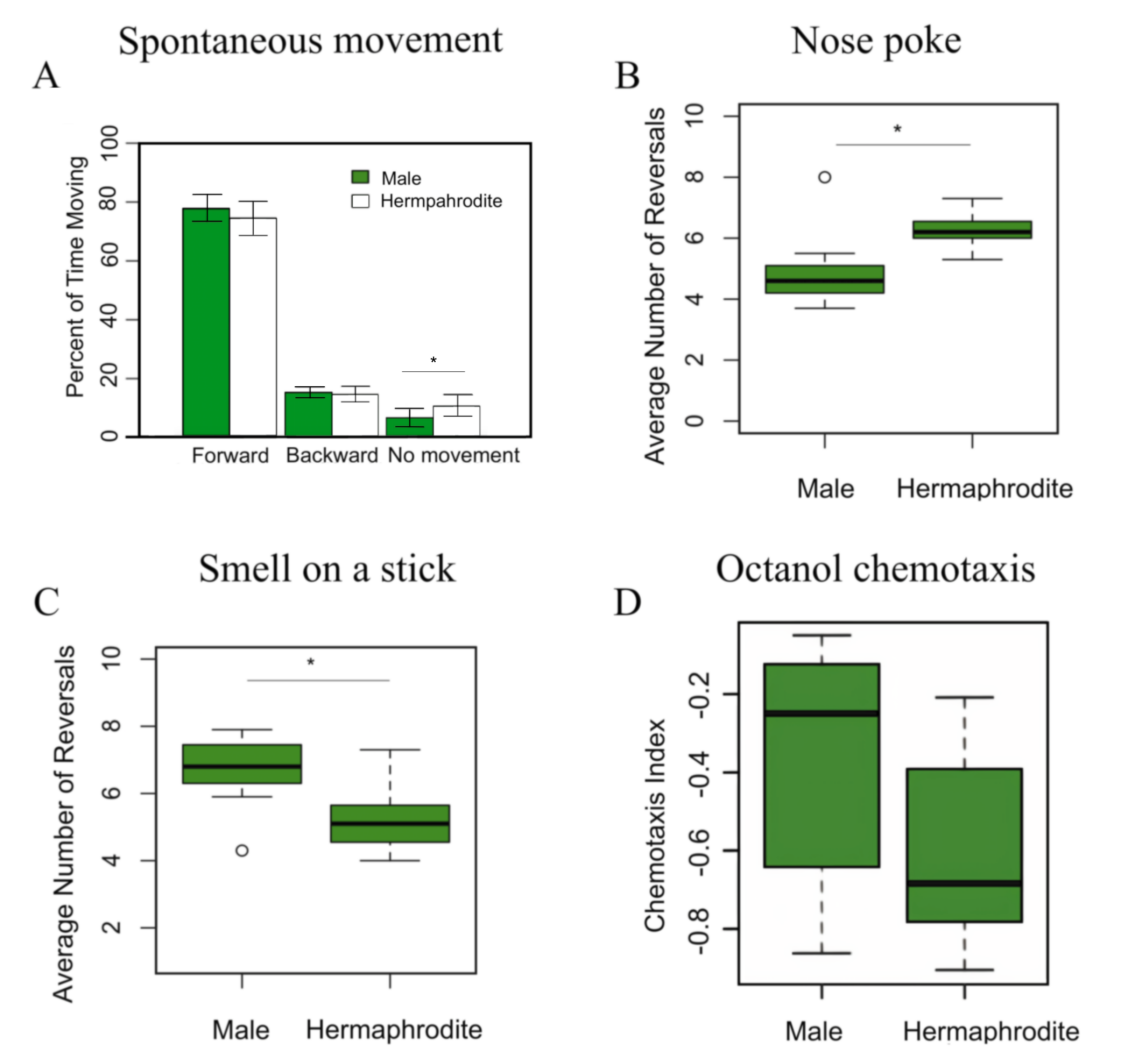
(A) **Males and hermaphrodites have little differences in their movement patterns.** The time that isolated worms spent moving forward, backward, or still was measured. No statistically significant difference was detected between the sexes in time spent moving forward (*p* > 0.05) or moving backward (*p* > 0.05). Males, on average, spent 77.6% of their time moving forward, while hermaphrodites spent an average of 74.3% of their time moving forward. On average, males spent 15.6% of their time moving backward, and hermaphrodites spent an average of 14.8%. Hermaphrodites spent significantly more time being still than males (*p* < 0.05), with an average time spent still of 10.8%, while males were, on average, still for 6.8% of the time. *n* = 140 worms, per sex. Error bars denote standard deviation. (B) **Hermaphrodites reverse more often to soft mechanical stimuli than males**. In response to encountering a soft mechanical stimulus (an eyelash tool), male *C. elegans* reversed 4.8 times out of 10, on average, and hermaphrodite *C. elegans* reversed 6.3 times out of 10, on average. This difference is statistically significant (*p* < 0.05). *n* = 15 trials, 10 worms per trial, for both sexes. (C) **Males reverse more often to exposure to high concentrations of butanone than hermaphrodites**. In response to an aversive smell stimulus, male *C. elegans* reversed 6.7 times out of 10, on average, and hermaphrodites reversed 5.2 times out of 10, on average. This difference is statistically significant (*p* < 0.05). *n* = 15 trials, 10 worms per trial, for both sexes. (D) **No statistical significance found between the chemotactic indices of male and hermaphrodite C. elegans.** Negative CI indicates avoidance of octanol. No significant difference in average CI of all males compared to all hermaphrodites. Male trial n = 10, hermaphrodite trial n = 10, average of 100 worms placed per trial.

## Description

*C. elegans* males and hermaphrodites differ genetically, physiologically, and neurologically. Hermaphrodites possess both X chromosomes (XX), while males have only one (XO) due to spontaneous nondisjunction that can occur during meiosis (Chasnov and Chow 2002). Males differ physiologically from hermaphrodites in that they are smaller, unable to produce eggs, and possess a blunted, fan-shaped tail, as opposed to the hermaphrodites’ long pointed tail. The males’ fan-shaped tail is used for cross-fertilization with hermaphrodites. In regards to their neural circuit, hermaphrodites possess 302 neurons while males have 385. Many of the males’ additional neurons are connected to the tail and are therefore used for mating. In addition, male *C. elegans* possess 91 sex-specific neurons, while hermaphrodites possess 8, and the remaining 294 neurons are not sex-specific (Portman 2007). Egg-laying, mate-searching, and pheromone-releasing behaviors have been of great interest to researchers (Barrios, Nurrish, and Emmons 2008). A handful of papers have presented rich studies regarding non-mating behaviors across the sexes, such as chemosensory behavior (Lee and Portman 2007), and locomotion (Mowrey, Bennett, and Portman 2014; Suo *et al.* 2019). We chose to focus on basic behavioral differences tests similar to those studied before as well as mechanosensation and another assay for chemosensation.

Considering the large number of sex-specific neurons present in male *C. elegans* -and a smaller number in hermaphrodites-, and the interconnected nature of neural circuitry, we proposed that aspects of behavior may differ between males and hermaphrodites in ways that are complementary to their differences in mate-seeking behavior. Males detect mates both through physical contact (Barrios, Nurrish, and Emmons 2008) and detection of pheromones (Simon and Sternberg 2002; White *et al.* 2007; Srinivasan *et al.* 2008). We hypothesized that males would spend more time spontaneously moving in reverse than hermaphrodites in an attempt to detect a mate with the copulatory process on the end of their tails. This takes into consideration the male copulatory mechanism that is located at the end of the male tail which, after coming into contact with another worm, drives worm movement in finding the hermaphrodite vulva (Liu and Sternberg 1995). Because the worms are sexually dimorphic (Lee and Portman 2007), we hypothesized that there would be a significant difference in sensitivity between males and hermaphrodites to mechanical and chemosensory stimulation that requires a quick and immediate response, but that they would perform comparably with stimuli that elicit slow and sustained reactions. We measured the responses to a nose poke and smell-on-a-stick behavioral assay, as well as a chemotaxis test. We were interested in examining differences in physical responses as previous studies have shown that males have a relatively higher locomotor activity and speed than hermaphrodites (Suo *et al.* 2019; Mowrey, Bennett, and Portman 2014).

The hypothesis that males would spend more time moving backwards than hermaphrodites was not supported by our results. Males and hermaphrodites spent equal amounts of time moving forward and backwards, although hermaphrodites spent significantly more time remaining still than males (Fig. 1A). However, the magnitude of difference of the time spent still between the sexes was not large, so it appears unlikely that any differences in spontaneous movement patterns between males and hermaphrodites would be biologically relevant.

The hypothesis that males and hermaphrodites would have differing sensitivity in immediate-response tasks was supported by our results, as the data showed that hermaphrodites reversed significantly more often than males after coming into contact with the eyelash tool (Fig. 1B). It is possible that because the eyelash tool is a soft stimulus with a width similar to that of a *C. elegans* worm, males may not have interpreted the eyelash as a foreign obstruction to be avoided, but instead a potential mate. A non-reversal response seen multiple times in males was a sliding motion alongside the eyelash tool, which may have indicated mate-seeking behavior (Portman 2007; Portman 2020).

The most interesting results are regarding the chemosensory abilities of males and hermaphrodites. We found that a high concentration of the typically attractive substance butanone (30% in ethanol) causes aversion, consistent with the fact that an attractant odorant can be aversive at high concentrations (Yoshida *et al.* 2012). We elected to use concentrated butanone as an aversive stimulus to avoid a possible ceiling effect present with strongly aversive odorants; thus, using butanone may allow for detection of differences that may not be possible with a very strong aversive stimulus. The results of the smell on a stick aversive smell stimulus assay showed that males reversed significantly more often than hermaphrodites after coming into close proximity with the butanone-ethanol solution (Fig. 1C). The results of the chemotaxis assay showed no significant difference between chemotactic indices of males and hermaphrodites (Fig. 1D). While our findings for the chemotaxis test were consistent with previous studies indicating a lack of significant differences, our smell on a stick results were novel in the differences they found.

There are a few possible reasons for the difference between the results of the chemotaxis and smell on a stick tests in terms of male sensitivity to aversive stimuli. Firstly, different aversive stimuli were used. Butanone is detected by the AWC neurons (Lee and Portman 2007) and octanol is detected by ASH neurons (Chao *et al.* 2004). Additionally, the aversive stimuli within the smell on a stick test requires immediate movement in a direction away from the toothpick, while repelling from a stimuli within chemotaxis elicits a slow behavioral response (Goodman 2006), which should also be taken into account when evaluating the data differences between the two tests. Olfaction is a complex neurological process, even in simple model organisms such as *C. elegans*, and multiple studies suggest that the olfactory process itself differs neurologically between male and hermaphrodite *C. elegans* (Lee and Portman 2007). Lee and Portman conducted a chemotaxis test as well as other behavioral assays to test olfaction in *C. elegans*, and the study found that male and hermaphrodite *C. elegans* display olfactory preferences, with sensitivity to smell stimuli varying depending on which specific chemosensory neurons are being stimulated as well as specific concentrations of the stimuli. Their overarching finding indicated that there is a discrepancy between male and hermaphrodite preference and sensitivity to different odorants. Thus, the increased reversals in males in the smell-on-a-stick assay could be due to the specific odorant, the rapidity of the test, or a combination of the two. We aimed to test a broad spectrum of behaviors between males and hermaphrodites to showcase that their behavioral differences might extend beyond their mating and reproductive behaviors.

## Methods

**Feeding conditions**

Worms were grown on maintenance plates containing NGM agar seeded with OP50 *E. coli*.

**Nose Poke**

Worms were individually picked to a 2 cm unseeded agar plate and left undisturbed for 2 minutes. An eyelash tool was placed in front of the head region during forward movement, and reversal responses to contact were recorded as a “yes” or “no”. Each worm was poked 10 times, with 5-10 second intervals between pokes. One trial consisted of 10 hermaphrodites and 10 males, and 15 trials were completed.

**Spontaneous Movement**

An individual worm was moved to an unseeded plate and left to acclimate for 3-5 minutes. The worm was then observed through a microscope for 2 minutes and its movements recorded- forward, backwards, or still movement. One trial consisted of recording 10 males and 10 hermaphrodites. 14 trials were completed.

**Smell on a Stick**

Worms were individually picked to a 2 cm unseeded agar plate and left undisturbed for 2 minutes. A toothpick dipped in a solution of 30% butanone in ethanol was placed in front of the head region during forward movement and no contact was made with the worm. Reversal responses to butanone were recorded as a “yes” or “no”. Each worm was tested 10 times, with 5-10 second intervals between exposures. One trial consisted of 10 hermaphrodites and 10 males, and 15 trials were completed.

**Chemotaxis**

Using a platinum wire pick, 100 males were picked to a seeded plate and then washed in M9 buffer and transferred using a glass Pasteur pipette to a micro-centrifuge tube. The worms were micro-centrifuged and washed three times then pipetted into the center of a plate divided into four quadrants with dots equidistant from the center. The test quadrants of this plate each contained 2 ul of 30% octanol and 2% sodium azide in ethanol solution on the dots, and control quadrants each contained 2 ul of 2% sodium azide in water and on the dots. After worms had been pipetted onto the plate, the plate was flipped and left undisturbed for one hour. Chemotactic index was calculated using the formula:

(# of worms in test quadrants – # of worms in control quadrants)

(total # of worms on plate – # of worms still in origin)

The procedure was then repeated with 100 hermaphrodites.

10 trials were performed on males and 11 were performed on hermaphrodites. Worms were excluded if they failed to exit the center circle.

**Statistics**

Student’s t-test was used to analyze significant differences between samples. Paired t-test was used for samples with identical n values, and equal variance t test was used for samples with different n values but similar variances.

## Reagents

N2 strains were provided by the CGC, which is funded by NIH Office of Research Infrastructure Programs (P40 OD010440). Mating pairs were used to create stocks enriched for males.
